# Development of a stereoscopic CT metal artifact management algorithm using gantry angle tilts for head and neck patients

**DOI:** 10.1002/acm2.12922

**Published:** 2020-06-07

**Authors:** Daniela Branco, Stephen Kry, Paige Taylor, John Rong, Xiaodong Zhang, Christine Peterson, Steven Frank, David Followill

**Affiliations:** ^1^ Department of Radiation Physics The University of Texas MD Anderson Cancer Center Houston TX USA; ^2^ Department of Imaging Physics The University of Texas MD Anderson Cancer Center Houston TX USA; ^3^ Department of Biostatistics The University of Texas MD Anderson Cancer Center Houston TX USA; ^4^ Department of Radiation Oncology The University of Texas MD Anderson Cancer Center Houston TX USA

**Keywords:** algorithm, head and neck, CT, metal artifact reduction, gantry angle

## Abstract

Dental amalgams are a common source of artifacts in head and neck (HN) images. Commercial artifact reduction techniques have been offered, but are substantially ineffectual at reducing artifacts from dental amalgams, can produce additional artifacts, provide inaccurate HU information, or require extensive computation time, and thus offer limited clinically utility. The goal of this work was to define and validate a novel algorithm and provide a phantom‐based testing as proof of principle. An initial clinical comparison to a vendor's current solution was also performed. The algorithm uses two‐angled CT scans in order to generate a single image set with minimal artifacts posterior to the metal implants. The algorithm was evaluated using a phantom simulating a HN patient with dental fillings. Baseline (no artifacts) geometrical measurements of the phantom were taken in the anterior–posterior, left–right, and superior–inferior directions and compared to the metal‐corrected images using our algorithm to evaluate possible distortion from application of the algorithm. Mean HU numbers were also compared between the baseline scan and corrected image sets. A similar analysis was performed on the vendor's algorithm for comparison. The algorithm developed in this work successfully preserved the image geometry and HU and corrected the CT metal artifacts in the region posterior to the metal. The average total distortion for all gantry angles in the AP, LR, and SI directions was 0.17, 0.12, and 0.14 mm, respectively. The HU measurements showed significant consistency throughout the different reconstructed images when compared to the baseline image sets. The vendor's algorithm also showed no geometrical distortion but performed inferiorly in the HU number analysis compared to our technique. Our novel metal artifact management algorithm, using CT gantry angle tilts, provides a promising technique for clinical management of metal artifacts from dental amalgam.

## 1 INTRODUCTION

Computed tomography (CT) imaging artifacts are discrepancies between Hounsfield unit (HU) values and actual linear attenuation coefficients of the object. These can pose a problem for physicians who are completing a diagnosis or attempting to identify or delineate the extent of disease. In the reconstruction of the CT images, dense objects such as bone and metal are a common source of artifacts through beam hardening and photon starvation. Beam hardening is caused by the presence of dense (and high atomic number) structures in the beam path. The x‐ray spectrum undergoes an upward shift in average energy due to the preferential attenuation of lower‐energy photons. CT reconstruction algorithms attempt to correct for beam hardening but are optimized for human tissues and cannot fully address highly attenuating materials such as metals.^1^ Photon starvation occurs when these highly attenuating materials cause the exiting x‐rays to have a low photon flux on the detectors. Consequently, the combination of beam hardening and photon starvation produces streaking artifacts in the reconstruction and can affect the image severely.

The most common artifacts present in head and neck (HN) cancer patients are the ones caused by the presence of high atomic number materials in the image, such as the ones originated by dental fillings. Dental filling metal amalgam artifacts can obscure the visualization of tumors in the oral cavity and oropharynx. This obscuring of the anatomy can lead to poor visualization of tissues and therefore improper definition of the target, potentially providing suboptimal management of the disease, particularly including radiotherapy quality. Studies have shown that the presence of dental artifacts can in fact increase the inter‐observer contouring variability of HN tumors.^2^ Aside from the difficulty in visualization of tumors and in definition of planning target volumes and organs at risk (OARs), metal artifacts will alter the true HU values in the affected voxels which negatively impacts the quality of radiotherapy in such areas. Kim et al. and Mail et al. have demonstrated that such artifacts result in increased dose heterogeneity and reduced target coverage.^3^, ^4^ In photon therapy, calculation errors were found to exceed 10% in an oral cavity clinical target volume when fillings are present, compared with 3% when no metal was present, emphasizing the potential severity of metal artifacts on dose calculations.^5^ The consequences for dose calculation accuracy are also particularly relevant in proton therapy because of the strong dependency between a correct relative linear stopping power prediction and accurate representation of HU values.^6^, ^7^, ^8^, ^9^ Proton treatment plans could display erroneous beam ranges and dose distributions when artifacts are present.

Several solutions for metal artifact reduction have been proposed, but many are impractical or not clinically feasible and therefore are not extensively adopted. Newhauser et al. accomplished a significant range uncertainty improvement in proton treatment planning with the use of megavoltage (MVCT) CT images.^10^ However, it is not known whether MVCT images can be used in treatment planning and their study was only performed for proton delivery of the pelvis area and has not been evaluated for other disease sites. Kim et al. recently published a post processing technique that requires an additional CT scan to obtain complementary image data to help reduce metal artifacts on the original scan.^11^ However, the success of their technique can be highly sensitive to noise and the severity of artifacts present. Replacing the metal amalgam in dental fillings with a radiologically inert composite material is also an available option. Composite fillings have been shown to demonstrate comparable HU ranges relative to native teeth.^12^ Nonetheless, filling replacements can take approximately 1 hr per tooth, and the cost and insurance coverage are not exactly determined, limiting the applicability of this technique. Current common practice to help manage metal artifacts includes the use of overriding techniques and of various metal artifact reduction algorithms. Overriding HU values in HN patients is the process in which dental fillings and their artifacts are identified, contoured, and assigned an expected HU value to be used in the dose calculation algorithms. Overriding HU for known or expected values in CT images is both time‐consuming and subjective, factors which can be eliminated with automated approaches such as available algorithms. The majority of current artifact reduction approaches involve algorithms that manipulate the raw projection data^13^, ^14^, ^15^, ^16^ and can generally be divided into two groups: iterative reconstruction algorithms and projection completions methods. The former approach starts from an initial guess image, re‐projects the image to the sinogram space, compares it to the original projections to generate a correction, applies that correction, and repeats that process until the difference between the images is minimized. This approach is superior at handling metal artifacts but it requires extensive computation time, making the technique clinically unfeasible. The latter approach works by replacing the corrupted projections in the sinogram space with interpolated data from regions of the sinogram unaffected by the metal. The estimation of the missing raw data values will determine how successful the algorithm is. Sharp transitions between the original projections and the interpolated ones cause additional artifacts. Moreover, the estimation of raw data values creates blurring in the final image due to data loss near the metal edges, which is not recoverable through the estimation of values. Despite the creation of additional artifacts and direct interpolation of HU information, projection completion methods gained more popularity. However, a recent study of three current commercially available artifact reduction methods concluded that they were generally not successful at reducing artifacts specifically caused by dental fillings.^17^ Indeed, particularly for dental artifacts, that study found that the commercial solutions had either a minimal effect or actually made the artifacts worse. Other post‐processing metal artifact reduction algorithms have been published but have not found clinical acceptance.^18^, ^19^, ^20^

Despite several publications of metal artifact reduction algorithms over the past two decades, there remains an evident need for better metal artifact management in highly heterogeneous sites, such as the HN.^5^, ^17^, ^21^ To address the need for better metal artifact management, we developed an algorithm that is not based on direct interpolation methods and therefore will not require the removal, replacement, and consequential loss of data points. In addition, the algorithm will not be system specific and thus could be used with any CT scanner that allows for gantry tilts, which is a feature offered on scanners from all major CT manufacturers. In this work, we will introduce the artifact management algorithm and provide testing on a geometrical phantom as a proof of principle. An initial clinical comparison to a vendor's current algorithm solution will also be presented.

## 2 MATERIALS AND METHODS

### 2.A Algorithm

**FIG. 1:**
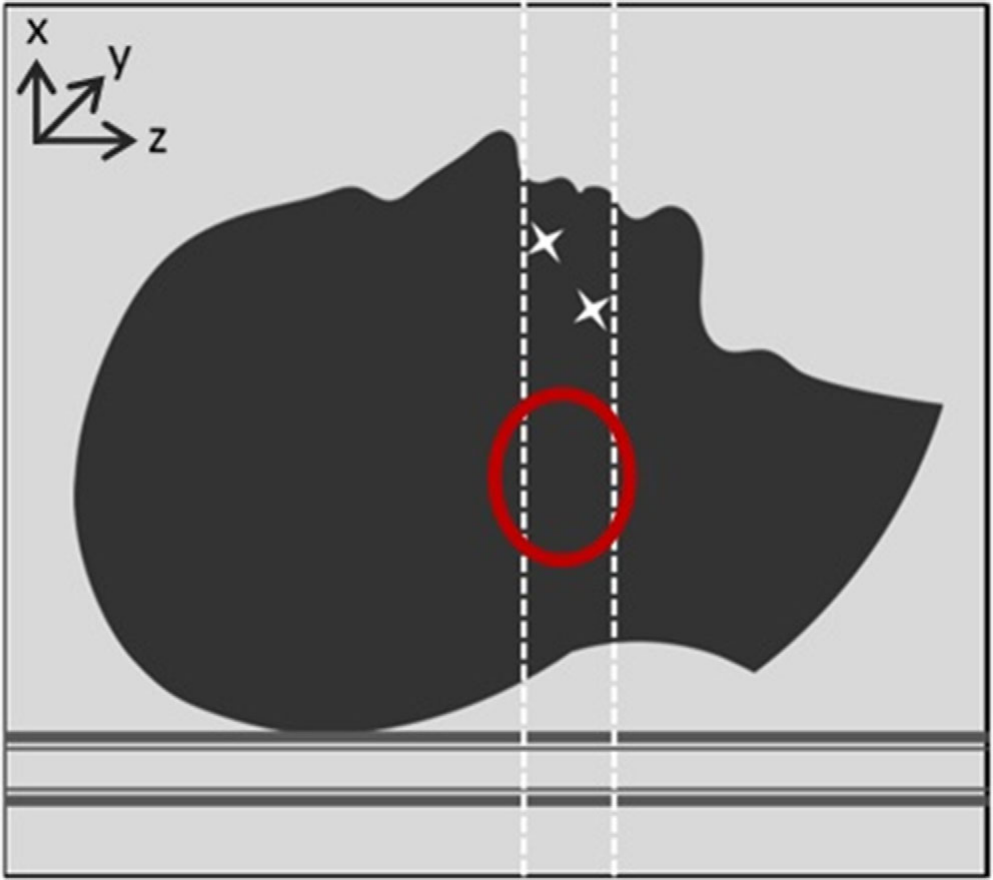
Diagram representing a patient with head and neck disease with the range of CT slices affected by the dental work. The red circle shows the region of typical head and neck disease that gets affected by artifacts resulting from metal in the mouth.

Similar to the concept of stereoscopic imaging, the algorithm developed in this work makes use of two angled CT scans to generate one artifact‐reduced image set. The issue with traditional 0° scans on patients with HN disease who have dental fillings or implants is that the artifact‐compromised slices are located where typical HN disease is located (Fig. 1), posterior to the oral cavity.

**FIG. 2:**
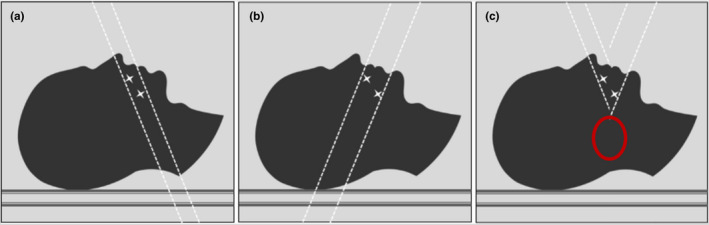
Diagram of a sagittal view of a head and neck patient with the CT gantry tilted superiorly (a) and inferiorly (b) showing the artifact‐affected slices. In the final artifact‐reduced image (c), the posterior region (red circle) is clear, and the artifacts are focused on the anterior region.

The goal of this algorithm was to use two‐angled CT scans to reconstruct an image where the posterior region can display the accurate HU information without the need for the widely used metal thresholding/deletion and interpolation techniques. In simple terms, the reconstruction technique is performed in the image space and is based on the combination of the superior portion of a superiorly tilted scan with the inferior portion of an inferiorly tilted scan, as shown in Fig. 2. The imaging technique and reconstruction algorithm create an image with the actual (not based on sinogram interpolation) HU information present in the posterior region of the patient's CT image. In order to remove the artifacts from the posterior region, the artifacts are focused on the anterior portion of the head. In other words, the artifacts now occupy previously unaffected regions such as the nose and chin, as it can be seen in Fig. 2(c). This trade‐off was made because disease and OARs typically are not located in those regions; the most common primary presentations of head and neck disease are the oropharynx and base of tongue. In the event of predominantly anterior disease, such as lip cancer, this trade‐off would likely not be appropriate and this approach may not be viable. However neoplasms arising in the nasal cavity and oral cavity are fairly uncommon, accounting for less than 1% and 2% of all HN cases in the United States, respectively.^22^

The framework for our new algorithm is divided into two main steps. Step 1 is responsible for untilting the images that were acquired at an angle, so that they appear as regular axial slices and can then be used in step 2. Step 2 uses those untilted images to form an image with the metal artifacts greatly reduced. The complete artifact reduction routine depends on the number of images per scan but on average takes less than 1 min to complete.

#### 2.A.1 Step 1: Untilting of image set

Step 1 was accomplished by performing three‐dimensional (3D) affine geometric transformations to the entire image volume. Affine transformations are done using linear mapping functions that preserve specific points, straight lines, and planes. After affine transformations, sets of parallel lines remain parallel, making them suitable for this part of the algorithm. In order to achieve such transformations, the algorithm performed a matrix multiplication with specific shear factors that were previously determined based on the angle of tilt of the gantry. The general form of this transformation is given by:

$$I=M\times I^{\prime }$$

where M is the geometric transformation matrix, I' the input image set, and I the final transformed image set:

$$I=\left[\begin{array}{c} x\\ y\\ z\\ 1 \end{array}\right],\;M=\left[\begin{array}{cccc} 1 & sh_{\mathrm{yx}} & sh_{\mathrm{zx}} & 0\\ sh_{\mathrm{xy}} & 1 & sh_{\mathrm{zy}} & 0\\ sh_{\mathrm{xz}} & sh_{\mathrm{yz}} & 1 & 0\\ 0 & 0 & 0 & 1 \end{array}\right],\;I^{\prime }=\left[\begin{array}{c} x^{\prime }\\ y^{\prime }\\ z^{\prime }\\ 1 \end{array}\right].$$

The first affine transformation was a shear transformation, and it was applied on the sagittal plane so that the tilted image volume could be resampled into the typical axial orientation. In order for that to happen, all shear factors in the shear transformation matrix M were set to 0, except *sh_zy_*. This factor determined the amount of shearing the image needed across the y‐axis and therefore was directly related to the CT gantry angle in which the image was acquired. The resampling of the tilted slices required an interpolation because of the possibility of the voxels being moved off the grid points while untilting the image set, and for this project, we used a linear interpolation. The second affine transformation performed on the image was a scaling transformation. Similar to the matrix multiplication done for the shearing transformation, the scaling was performed by applying the appropriate factors on the M matrix (Table 1), which were determined empirically to provide the best match between the reconstructed and baseline images. CT images acquired at an angle are elongated on the y‐axis and therefore need a correction along that direction. For our setup, appropriate scaling was obtained when all elements of the identity matrix M were set to 0 except the position *M_2,2_*, which scales the y‐axis. This process was completed for the superior and inferior tilted images so that the new transformed image sets were then displayed as traditional axial slices and could be used in step 2.

**TABLE 1:** Shear and scaling factors used in the affine transformations.

Angle	Shear factor	Scaling factor
5˚	0.019	0.999
10˚	0.041	0.989
15˚	0.060	0.965
20˚	0.078	0.945
25˚	0.092	0.905
30˚	0.108	0.871

#### 2.A.2 Step 2: Correction of metal artifact

The superior and inferior modified image sets from step 1 were used to reconstruct the final artifact‐reduced image. On each image set, this portion of the algorithm searched axially through each pixel, starting at the superior‐most slice, until it found the first metal thresholded pixel (above 2000 HU). That slice number was saved, and the same process was performed in opposite order starting from the inferior‐most slice. This repetition was done to determine the first and last slices on which artifacts were present. The number of slices with artifacts is dependent on the size of the implants and slice thickness, but for this phantom, the images contained approximately 2.0 cm (in the superior–inferior direction) of artifact‐affected slices. Once those were identified, a center slice (in the center of the artifact‐affected slices) was determined by averaging the first and last slice numbers previously determined and used as the reference slice for that particular image set. The artifact‐free slices were selected in each image set, up to the reference slice (e.g., the superior slices up to the reference slice in the superiorly tilted image set and the inferior slices up to the reference slice in the inferiorly tilted image set). Finally, the artifact‐free slices were merged as shown in Fig. 2(c), and the final artifact‐reduced image was formed. In addition, the metadata in the final untilted image volume created required a correction. The reconstructed images were still assigned an angled metadata tag and had to be corrected in order to be properly displayed in the imaging software and treatment planning systems. Clinical implementation of the algorithm could entail the scanner console having AMPP installed so that once both images are acquired the final artifact‐reduced image can be reconstructed and displayed for physician approval. If vendor agreements cannot be established to have the AMPP protocol installed along with their software, both image sets can be extracted, and post processed at an office computer at the institution.

### 2.B Evaluation

**FIG. 3:**
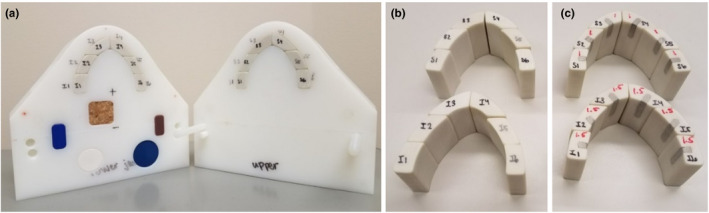
Both halves of the geometrical phantom (a) and the tooth structures without (b) and with (c) metal amalgam fillings used in the testing of the algorithm.

The routine described in the previous section was tested on a geometrical phantom simulating a HN cancer patient with dental fillings. The phantom was composed of high‐impact polystyrene and was divided into two halves representing an upper and lower human jaw so that teeth structures could be inserted [Fig. 3(a)]. The tooth structures were made of Gammex 450 cortical bone substitute (Middleton, WI). Two sets of teeth were used in the evaluation of the algorithm. The first one was used as the baseline and contained only the cortical bone material in order to obtain the artifact‐free image set [Fig. 3(b)]. The second set was modified to contain Dispersalloy (Dentsply, Milford, DE) dental amalgam (physical density = 9.6 g/cm^3^), as seen in Fig. 3(c). The general configuration and size of the dental fillings inserted in the modified set were determined by a dentist. The phantom was 15 cm × 13.5 cm × 8 cm, and each tooth was approximately 3 cm long. The modified set of teeth contains 1.5 cm long metal amalgam fillings.

The geometrical phantom also contained plugs of different materials located posterior to the teeth set; these materials were selected to span the range of HU values seen in images of HN patients. These plugs were two lateral rectangles made of solid water (1 cm × 2 cm × 2 cm, physical density = 1.04 g/cm^3^; CNMC, Nashville, TN, USA) and blue water (1 cm × 2 cm × 1 cm, physical density = 1.09 g/cm^3^; Gammex, Middleton, WI, USA), two cylinders made of polybutylene terephthalate (PBT) (dimeter: 2.5 cm × 1 cm, physical density = 1.31 g/cm^3^; Gammex, Middleton, WI, USA) and Techtron HPV bearing grade (dimeter: 2 cm × 1 cm, physical density = 1.430 g/cm^3^; Gammex, Middleton, WI, USA), and a cuboid made of cork (2 cm × 2 cm × 4 cm, physical density = 0.24 g/cm^3^). The plugs were used as structures of interest in the phantom to help test the integrity of the algorithm and its ability to remove artifacts. Geometrical distortion and HU accuracy measurements were used to establish a comparison between the metal‐free scan (baseline) and the reconstructed image set.

To quantify the algorithm performance, geometric distortion was evaluated through geometrical measurements of the plugs and phantom taken in the anterior–posterior (AP), left–right (LR), and superior–inferior (SI) directions. Measurements were done with the CT gantry at 0° with no metal teeth (baseline) and with metal amalgam (eight total metal teeth) and at the six gantry tilt angle reconstructions performed (5°, 10°, 15°, 20°, 25°, and 30°). All scans in the algorithm performance analysis were scanned on the GE Lightspeed VCT using the HN CT protocol and the parameters are listed in Table 2. To maintain consistency throughout all the measurements, the plug dimensions were obtained by measuring the full width at half maximum (FWHM) on HU profiles across the center of each plug. HU accuracy testing was done measuring fixed region of interest (ROI) sizes among the different reconstructed scans. Mean HU numbers were collected inside each plug and compared between the baseline scan and reconstructed image set with reduced artifacts. All measurements were done using Eclipse treatment planning system (version 13.6; Varian Medical Systems, Inc., Palo Alto, CA, USA). In addition to the algorithm performance analysis, the technique developed in this work was compared to the vendor's artifact reduction algorithm solution; SmartMAR. For that scan, the phantom was imaged with the metal teeth in place and reconstructed with SmartMAR applied. Similar to the previous analysis, geometrical and HU number measurements were obtained from the phantom's metal scan and compared to baseline images obtained from the same scanner (Table 2).

**TABLE 2:** Imaging protocols for all scans obtained.

	kVp	mAs	Slice thickness (mm)	Filter type	Recon kernel	Tube rotation time (s)
Angle technique (all angles)	120	400	2.5	medium filter	Standard	0.8
Baseline	120	400	2.5	Medium filter	Standard	0.8
Metal uncorrected	120	400	2.5	Medium filter	Standard	0.8
Baseline – SmartMAR	120	320	3.75	Medium filter	Standard	1.0
Metal Corrected – SmartMAR	120	320	3.75	Medium filter	Standard	1.0

## 3 RESULTS

### 3.A Qualitative analysis: Artifact removal attainment

**FIG. 4:**
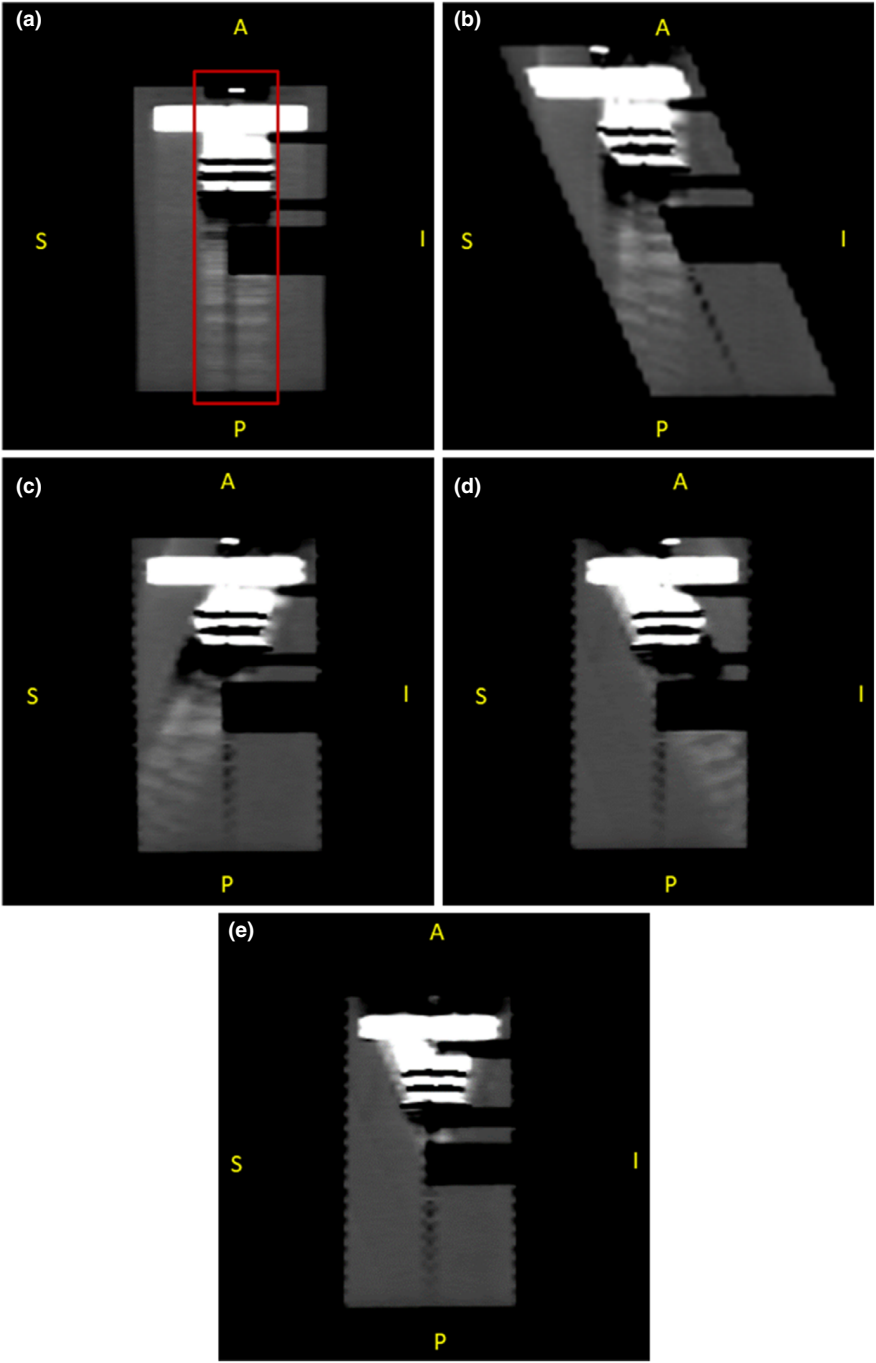
(a) Sagittal view of the uncorrected image set of the geometrical phantom acquired at a 0° angle with metal teeth. The box indicates artifact‐affected slices. (b) Uncorrected image acquired at a 25° angle appearing tilted and elongated. Inferior tilted scan (c) and superior tilted scan (d) after the first part of the algorithm showing the desired typical axial appearance and corrected to normal height. (e) Final artifact‐reduced image reconstructed using (c) and (d).

The first step of the algorithm developed in this work successfully untilted and corrected the angled CT image set. Figure 4(a) shows a sagittal screenshot of the 0° scan of the geometrical phantom with the metal teeth inserted and the artifacts created by them. As expected, the artifacts extended all the way through the posterior region of the phantom. Figure 4(b) demonstrates how the images acquired at an angle appear tilted and elongated. This particular example is a superior 25° tilt and shows the artifacts running perpendicular to the couch along the AP direction. After the first part of the algorithm was completed, the image set [Fig. 4(c)] showed the two halves of the phantom and its structures as it would be seen in the regular perpendicular scan [Fig. 4(a)], except with the artifacts extending away from the HN posterior region of interest. Figure 4(d) shows the end result for the inferiorly tilted image set. It is important to notice the geometric distortion along the AP direction in the uncorrected image set [Fig. 4(b)]. The uncorrected images acquired at an angle become elongated (in the AP direction only) compared with the real phantom size. Figures 4(c) and 4(d) show the phantom after the algorithm was applied, corrected to normal height and untilted, which were later used to form Fig. 4(e) showing the final artifact‐reduced reconstructed image set.

**FIG. 5:**
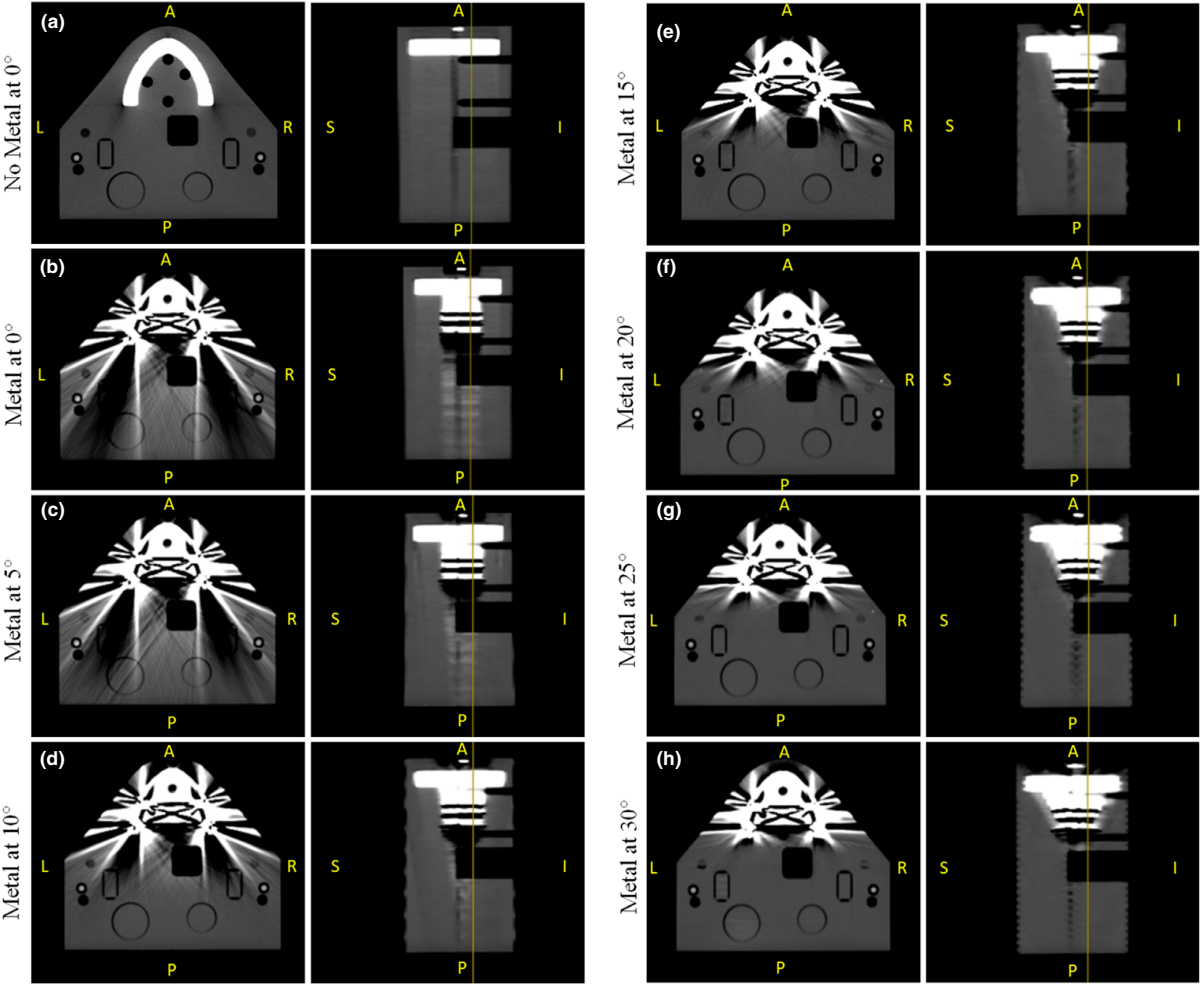
Axial and sagittal views of the geometrical phantom after the complete algorithm was applied. Images show the phantom with (a) no metal at 0°, (b) metal at 0°, (c) metal at 5°, (d) metal at 10°, (e) metal at 15°, (f) metal at 20°, (g) metal at 25°, (h) metal at 30°.

The metal artifact reduction step of the algorithm design was successful at managing artifacts in the posterior region of the phantom. This can be seen in Fig. 5, which shows the axial and sagittal views of the geometrical phantom at the same slice location for each CT angle examined. The vertical yellow line on each sagittal view represents the corresponding axial image. It is possible to see that as the CT gantry angle increases, the posterior region of the artifacts created by the metal amalgam becomes clearer. As mentioned in the previous section, the artifacts in the reconstructed images remained in the mouth and chin regions, which can be seen more clearly on the larger angle reconstructions.

### 3.B Quantitative analysis: Algorithm integrity analysis

**FIG. 6:**
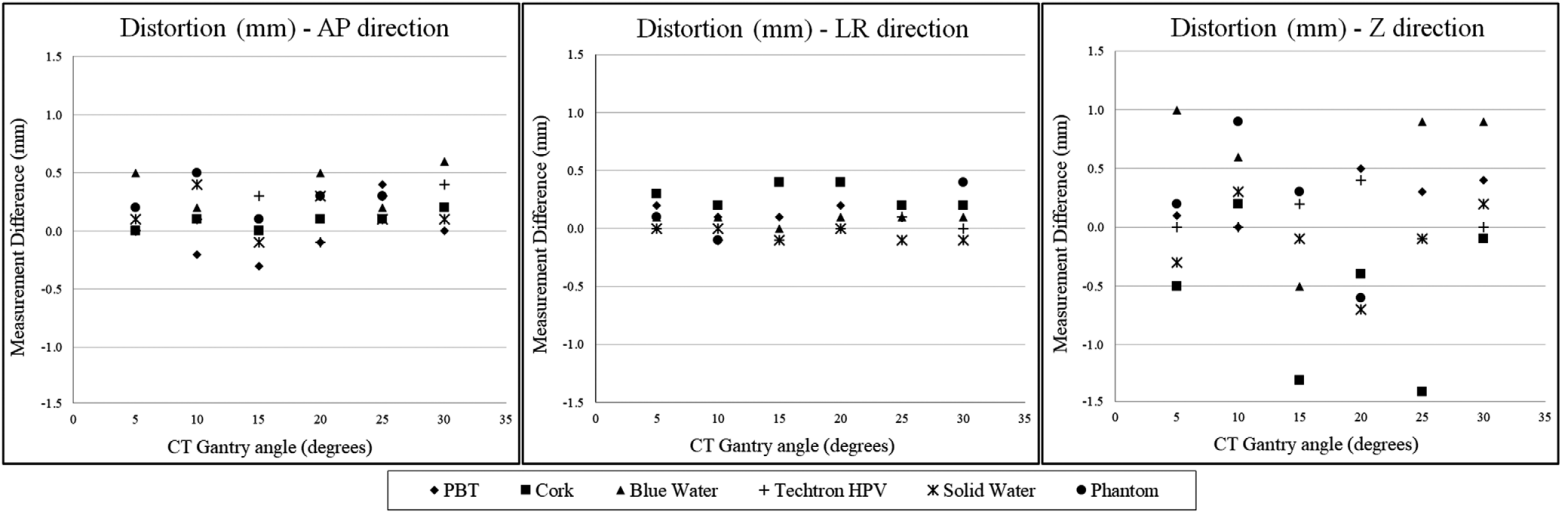
Results for distortion measurements showing each difference obtained between the measurements of the different plugs and phantom done with the gantry at 0° with no metal teeth present (baseline) and with metal teeth present for the six different CT gantry angles in the AP (a), LR (b), and SI (c) directions.

In addition to the qualitative examination of the algorithm, the distortion measurements are shown in Fig. 6. Each data point represents the difference between the measurements of the plugs and phantom with the gantry at 0° with no metal teeth present (baseline) and with metal teeth present, for the six CT gantry angles. It is possible to see that there is no trend in the measurements across the different gantry angles and different directions, indicating that our artifact management algorithm will provide geometrically accurate images for any CT gantry angles used. Owing to the fact that no trend was observed, total averages were calculated for all the plugs and phantom measurements in each direction. The average total distortions for all gantry angles in the AP, LR, and SI directions were 0.17, 0.12, and − 0.14 mm, respectively, and no statistical correlation between the spread of the results and the gantry tilt utilized for the algorithm was observed. A negative data point on the plot means that the distance measurement of the plug on the corrected image set were smaller than on the baseline image set. To test the reproducibility of the measurement technique, the standard deviation (SD) was calculated for the lowest‐ and highest‐density sample materials: cork and Techtron HPV. For each direction, 10 FWHM measurements were obtained and showed an average SD between the materials of 0.12, 0.13, and 0.33 mm for the AP, LR, and SI directions, respectively.

The measurement data for each material were fitted with a linear regression line and analyzed for statistical significance in which the dependent variable was each material in question. An α level of 0.05 was used; all *P*‐values calculated for the materials’ regression lines’ slopes showed no significance, indicating there was no distortion pattern related to gantry angle. The fitted slopes varied from positive to negative with very small values, ranging from −0.05 to 0.014, again indicating no trend. In addition, all regression line intercepts included 0 in the 95% confident intervals (CIs), showing no evidence that the intercepts of the linear fit were positive or negative. The statistical analysis performed here showed that the measured geometrical distortion was random and not correlated with CT gantry angle.

**FIG. 7:**
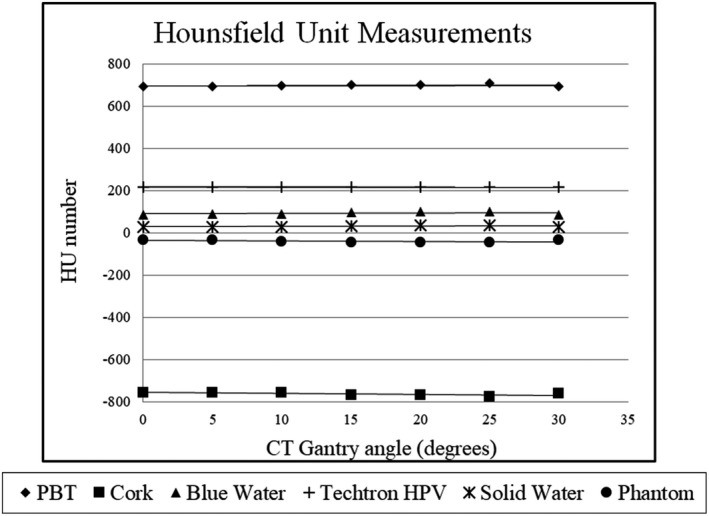
Hounsfield unit (HU) measurements of all materials for the different CT gantry angles.

The HU measurements also showed no correlation with varying gantry angles and are shown in Fig. 7. Linear regression lines were also fitted for each material, and the same regression analysis was performed as described above. Similar to the distortion measurements, all of the material slopes’ *P*‐values were above significance level, indicating no pattern correlating HU with gantry angle tilt. Additionally, for each material, the HU values measured were statistically consistent (within the 95% CI) with the true (untilted) HU value.

### 3.C Comparison with commercial solution

**FIG. 8:**
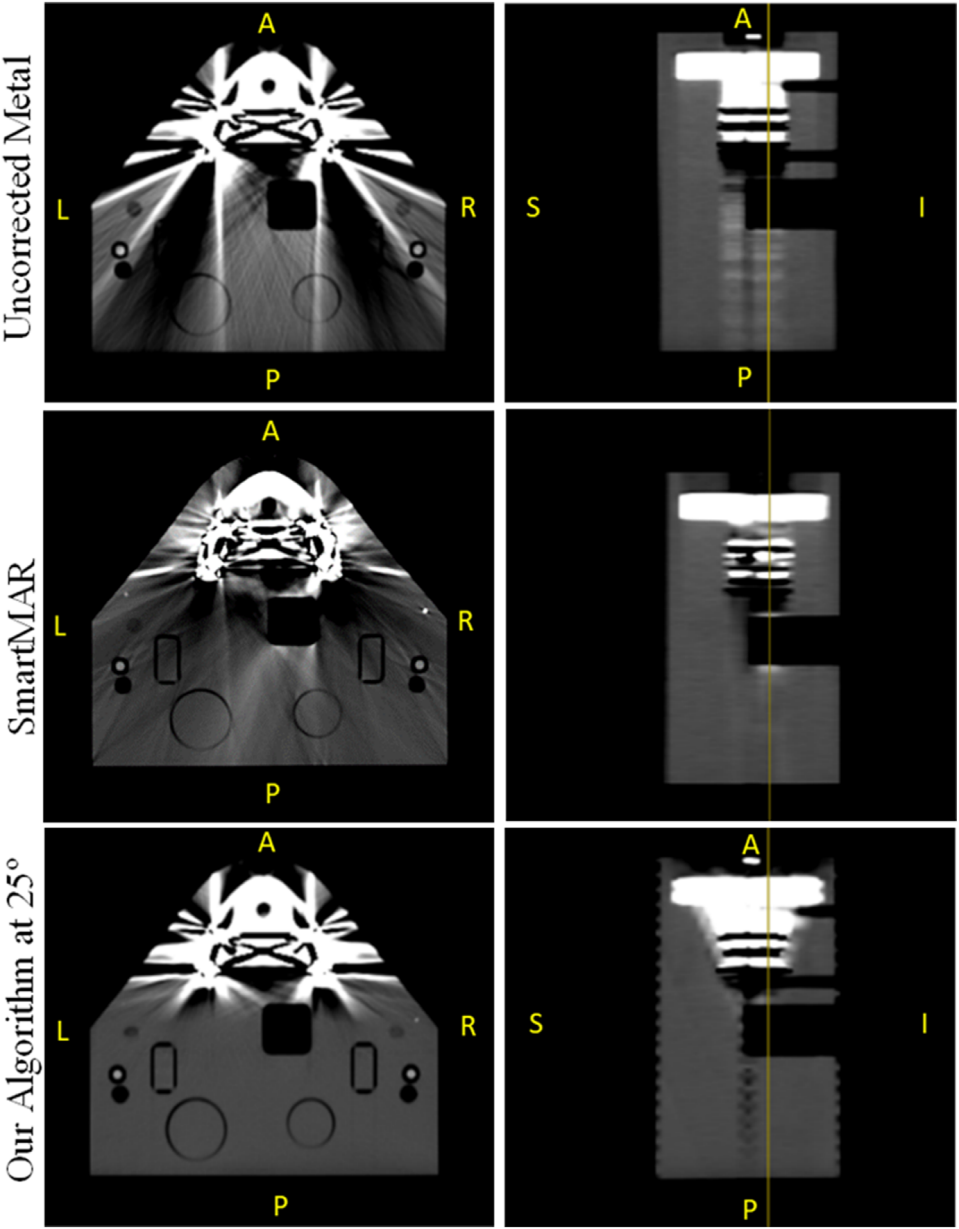
Side‐by‐side comparison of SmartMAR and the technique developed here to the uncorrected metal scan of the phantom.

Figure 8 shows a side‐by‐side comparison of the same slice in the phantom after our artifact management technique and SmartMAR were applied. Qualitatively, it is possible to see that SmartMAR improves part of the streaking but creates other artifacts in the image. It is also noticeable that SmartMAR affects the entire slice in which metal is present. That is due to the algorithm being performed in the sinogram space. In contrast, our algorithm creates no additional artifacts in the posterior region and fully eliminates the streaking caused by the metal implants.

In the quantitative comparison between both techniques, it was discovered that SmartMAR corrected images showed no relevant geometrical distortion. The average total distortions for all plugs and phantom in the AP, LR, and SI directions were very small, 0.03, 0.05, and −0.3 mm, respectively. Table 3 shows the HU numbers collected and their standard deviations (SD) inside the fixed ROIs for all scans. Table 4 shows the HU number differences between the relevant scans; metal uncorrected and respective baseline, and metal corrected (SmartMAR and our Technique) and respective baselines. Ideally the HU number difference should be close to 0, indicating no difference between the baseline scan and the corrected image set in question. However, the metal uncorrected scans are filled with dark and light streaks hence showing large HU differences. The streaking is also indirectly represented by the SD inside the ROIs (Table 3); the uncorrected image sets showed large variations in HU numbers, hence displaying large SD values. It is possible to notice that, for most plugs (blue water, Techtron HPV, solid water, and phantom), SmartMAR provided some improvement in the HU accuracy with HU differences closer to 0 then the metal uncorrected images. However, the HU number values were still notably different from what they were expected to be (baseline). It is important to notice that the PBT and cork plugs’ HU values were worsened by SmartMAR's algorithm. In contrast, our technique showed very small differences in HU number, indicating that the image quality in the posterior region of the phantom was nearly identical to the scan without any metal present. In addition, due to the absence of streaking in the posterior region of the phantom, the SD values collected on the scan corrected with our algorithm were much smaller than SmartMAR's and comparable to the baseline values (Table 3).

**TABLE 3:** HU numbers and standard deviations in parenthesis of plugs and phantom for all relevant scans.

Structure	HU number Mean			
	Baseline	Metal uncorrected	SmartMAR corrected	Our technique at 25˚ corrected
PBT	693 (2)	681 (31)	683 (8)	695 (1)
Cork	−758 (10)	−657 (27)	−496 (15)	−756 (12)
Blue Water	87 (0)	−70 (28)	45 (2)	91 (1)
Techtron HPV	218 (1)	306 (57)	167 (21)	216 (1)
Solid Water	29 (1)	−113 (19)	−34 (4)	29 (2)
Phantom	−36 (2)	19 (9)	−5 (8)	−40 (1)

**TABLE 4:** HU number differences between the relevant scans for both metal artifact reduction techniques.

Structure	HU number difference with baseline (HU)		
	Metal uncorrected	SmartMAR	Our technique at 25˚
PBT	−13	21	−1
Cork	−109	280	−2
Blue Water	171	40	−4
Techtron HPV	−89	50	2
Solid Water	153	55	0
Phantom	−66	35	5

## 4 DISCUSSION

The algorithm generated in this work was successful at eliminating metal artifacts created by dental amalgam in the posterior region of the image. It is possible to see that as the CT gantry angle increases, the posterior region becomes clearer of metal‐affected pixels, leading to better visualization of the structures in the phantom. As a consequence of the combination of two angled scans, the artifacts extend to regions that were previously unaffected, such as the nose and chin. However, as previously mentioned, those areas do not normally contain disease or OARs.

Several metal artifact reduction methods have been proposed and are currently available to the community. These methods include the use of MVCT, dual‐energy CT, magnetic resonance imaging, additional CT scans, and the complete removal of the dental work. However, each has important limitations and hence lacks wide clinical acceptance. A common technique in radiation oncology is to manually override HU values, but this has major drawbacks in that anatomy is still obscured and is now assumed to be homogeneous. Current metal artifact reduction algorithms are promising but have the downside of replacing missing data with artificially interpolated generated data. That approach creates additional uncertainty in the HU information, and such uncertainty is undesirable in diagnosis and in therapeutic dose calculations, particularly in applications such as proton therapy.

The metal artifact reduction technique presented in this work uses two angled CT scans to eliminate the metal artifacts posterior to the dental implants and produce accurate and faithful HU number information. Unlike the existing algorithms, the one developed here uses the correct HU information to reconstruct the final image and does not rely on metal thresholded sinogram deletions and interpolation of data, which can cause more artifacts^21^ and uncertainty in HU accuracy. The technique developed in this study also has the potential to be applied to other areas of the body that have metal inserts (e.g., surgical clips, prostheses) and therefore has the potential for improvement in artifact management and imaging of anatomical structures other than just HN. Another advantage of this technique is that it is performed in the image space and hence can be used by any CT scanner vendor. Raw data are intellectual property proprietary to each vendor and thus, is very difficult to attain. The method developed here does not require the manipulation of raw data and therefore can provide images with minimal artifacts posterior to the oral cavity.

The size difference measurements for all the plugs showed no correlation with gantry angle, in all directions. Angled CT scans elongate the imaged object in the AP direction, but these distortions were managed using a geometrical correction applied in the first part of the algorithm. Following that correction, all distortion measurements were on the order of 0.1 mm, with similarly small SDs. Measuring the distances in the SI direction was more challenging owing to worse resolution in that direction, yielding a larger observed SD—nearly three times that seen in the AP and LR directions. Similar to the distortion findings, the HU number was not correlated with gantry angle and showed high consistency throughout the different reconstructed images compared with the baseline image set. These results indicated that our novel technique described here provided nearly artifact‐free images in the posterior region of the phantom that were geometrically and HU number accurate when compared to a metal‐free baseline. In addition to being geometrically and HU number accurate, it also outperformed a current commercially available artifact reduction method. When compared to SmartMAR, our technique provided better HU accuracy in the posterior region of the phantom and corrected the streaking caused by the metal implants better, shown by the improved HU difference and SD measurements inside the plugs and phantom.

The technique developed here manages metal artifacts with the use of correct (not interpolated or manipulated) data but carries one potential drawback. The reconstruction of the artifact‐free image requires two scans instead of one, which may deliver additional dose to the patient. The cost of any extra dose (<10 cGy) would need to be weighed against improved image quality and associated benefit to diagnosis and/or radiotherapy treatment of patients. The comparison to SmartMAR shown in this manuscript is indicative of the potential benefit behind our approach. For radiotherapy patients in particular, this one‐time extra dose is negligible (< 0.1%) compared with the total treatment and imaging dose already committed to HN cancer patients.

The presented methodology in this manuscript was the introduction of a stereoscopic solution to reduce metal artifacts present in patients’ HN CT images through the use of CT gantry angles. Future work will expand the image quality and robustness study (effects of head tilt, slice thickness, etc) comparing this technique to all major vendors’ current metal artifact reduction algorithms with the use of a HN anthropomorphic phantom. We will also further investigate the performance of the technique in the context of radiation therapy and treatment planning system dose calculations. A dosimetric analysis will be performed on the anthropomorphic phantom to show the advantages of the algorithm developed here over the other approaches currently in use. Proton treatment planning dose calculations and proton beam differences will be of particular interest because of their large dependency on HU accuracy and robustness.

## 5 CONCLUSION

A stereoscopic metal artifact management algorithm was developed using CT gantry angle tilts and evaluated in a geometrical HN phantom. The algorithm developed here offered the improvement of not requiring the replacement of deleted metal thresholded data with artificially interpolated data. In addition, it used accurate HU data obtained from two different scans and was divided into two parts: the first included the untilting and correction of the angled image set, and the second involved the removal of the metal artifacts in the posterior region. Unlike other existing algorithms, this algorithm is independent of the CT scanner provider and therefore can be used in any scanner that allows for gantry tilts. Also, our technique is applied in the image space, and therefore does not require the need to acquire and manipulate the proprietary raw data from vendors. The images showed the successful removal of the artifacts present in the posterior region on the phantom, allowing for much better visualization of the structures. The quantitative analysis of the algorithm performance showed that it presents artifact corrected images with no geometrical distortion and with HU number accuracy when compared to the baseline. In addition, our technique outperformed a commercially used algorithm, SmartMAR, in providing artifact‐free images with better HU agreement with the metal‐free baseline. Future work will be done to further expand the image quality analysis and robustness among all major vendors’ solutions, and to evaluate treatment planning dosimetry, specifically applied to proton therapy since proton treatment quality and robustness are highly dependent on HU accuracy.

## CONFLICT OF INTERESTS

The authors have no relevant conflicts of interest to disclose.
